# Prescription Patterns of Asthma Preventers Among Children and Adolescents Between Australia and South Korea

**DOI:** 10.3389/fphar.2022.834116

**Published:** 2022-05-20

**Authors:** Min Sook Seo, Jodie Hillen, Dong Yoon Kang, Nicole Pratt, Ju-Young Shin

**Affiliations:** ^1^ School of Pharmacy, Sungkyunkwan University, Suwon, South Korea; ^2^ Quality Use of Medicines and Pharmacy Research Centre, Clinical and Health Sciences, University of South Australia, Adelaide, SA, Australia; ^3^ Department of Preventive Medicine, Ulsan University Hospital, Ulsan, South Korea; ^4^ Samsung Advanced Institute for Health Sciences and Technology, Sungkyunkwan University, Seoul, South Korea; ^5^ Department of Biohealth Regulatory Science, Sungkyunkwan University, Seoul, South Korea

**Keywords:** asthma preventers, paediatric patients, ICSs, LTRAs, Australia, South Korea

## Abstract

**Purpose:** Inhaled Corticosteroids (ICSs) and oral Leukotriene Receptor Antagonists (LTRAs) are commonly prescribed asthma preventers, however, concerns have been raised as to whether montelukast (LTRA) is associated with an increase in occurrences of neuropsychiatric side effects in children. Our study was conducted to observe prescribing patterns of asthma preventers among paediatric patients specifically focusing on ICSs and LTRAs between Australia and South Korea to see intercountry differences in the use of these medicines.

**Materials and Methods:** The Health Insurance Review and Assessment Paediatric Patients Sample dataset for South Korea and data provided by Services Australia were used in the study. Paediatric patients aged between 3 and 19 with more than one dispensing of an asthma preventer and at least one reliever between 1 Jan 2018 and 31 December 2018 were selected. Prevalence per 1,00,000 persons and standardised prevalence were estimated.

**Results:** A total of 3,58,470 patients (2,04,270 from South Korea and 1,54,200 from Australia) were included in the study. A higher prevalence of ICS-based inhalers was seen in Australia with 80.1% compared to 13.5% in South Korea. In addition, Australia showed a stronger tendency of prescribing high dose ICS-based inhalers compared to South Korea with 22.9% vs. 4.9%. In contrast, use of LTRAs was more prevalent in South Korea with 57.6% while in Australia, montelukast was the only LTRA dispensed at a proportion of 18.9%. Moreover, 29.9% of xanthines which are orally available preventers, were prescribed more frequently in South Korea compared to Australia (0.1%).

**Conclusion:** Australia showed a tendency of prescribing ICS-based preventers whereas South Korea exhibited a preference towards the oral LTRAs. Given the potential risk of neuropsychiatric side effects among paediatric patients with montelukast, reasons for the high use of montelukast in South Korea should be investigated further.

## Introduction

Asthma is a chronic inflammatory disease of the airways affecting approximately 235 million people worldwide and it is one of the most prevalent medical conditions in childhood and adolescence (Global Asthma Network, 2018; Sol et al., 2019). English-speaking parts of the world such as Australia, the United Kingdom and North America have the highest prevalence of asthma with more than 20% diagnosed with the condition. Australia has been recognised as having a relatively high prevalence of asthma in young people (>20%) compared to Asia-pacific countries which report a prevalence of <5% (Lai et al., 2009; Global Asthma Network, 2018; Sol et al., 2019). Asthma in childhood is considered a major burden on health care resources due to the high number of hospital visits (Lai et al., 2009; Global Asthma Network, 2018; Sol et al., 2019). In 2016, asthma contributed 23.7 million Disability Adjusted Life Years (DALYs) across all ages, ranking 28th among the leading causes of burden of disease (Global Asthma Network, 2018).

The mainstay of treatment is the use of relievers (to relax the bronchial smooth muscle and improve airflow) and preventers (to prevent long-term inflammation and exacerbations) (Lai et al., 2009; Global Asthma Network, 2018; Sol et al., 2019; Australian Medicines Handbook, 2020). According to clinical guidelines in South Korea and Australia, paediatric patients requiring a preventer can be prescribed either inhaled corticosteroids (ICSs), leukotriene receptor antagonists (LTRAs), inhaled corticosteroids and long acting beta_2_ agonists combination (ICS + LABA), cromones, sustained release xanthines and/or biologics depending on the severity of their asthma (Masoli et al., 2004; GINA, 2015; GINA, 2019; Australian Medicines Handbook, 2020; Ertoy Karagol and Bakirtas, 2021).

International guidelines recommend either ICSs or LTRAs as first line treatment however, systematic reviews differ in their recommendations depending on the country of origin. Systematic reviews conducted in the United States and Italy suggested use of ICS as the 1st line preventer treatment in children whereas reviews performed in Hong Kong and Sri Lanka found that LTRAs are equally effective as ICS, thus could be used as the 1st line preventer (Hon et al., 2014; Massigham and Smaldone, 2014; Hossny et al., 2016; Jayawardena et al., 2019). An advantage of LTRAs over ICS products is that they are oral preparations compared to inhalation. Inhalation of medication often requires education and regular review for appropriate administering techniques as young children can find the techniques difficult to master (Choi et al., 2017; Choi et al., 2018; Australian Medicines Handbook, 2020).

Whilst all preventer medications have Adverse Drug Reactions (ADRs), montelukast has been associated with serious neuropsychiatric ADRs such as agitation, depression, anxiety, suicidal thoughts and actions. In 2020 the FDA included serious neuropsychiatric ADRs as a black box warning on montelukast packaging (Hon et al., 2014; Choi et al., 2017; Australian Medicines Handbook, 2020; Drug Safety Communications, 2020). Given the different modes of administration, guidelines and ADR profiles, we undertook this study to compare and contrast utilisation patterns of these preventative medicines between Australia and South Korea. Utilisation patterns will help to inform future studies of safety and effectiveness of preventer medications in the paediatric population.

## Materials and Methods

### Data Source

The data for Australia were provided by Services Australia (http://www.serviceaustralia.gov.au/organisations/about-us/statistical-information-and-data) and the claims data of the Health Insurance Review and Assessment Service (HIRA) for South Korea were used for the study. The study period was from 1st January 2018 till 31st December 2018. This is a standardised, longitudinal, unit-record extract encompassing all medicines dispensed under the Pharmaceutical Benefits Scheme (PBS) for a random 10% sample of Australians which is approximately 2.5 million people (Mellish et al., 2015). The claims data of HIRA are accrued by reimbursements for services provided by health care professionals to patients and consist of 10% of inpatients and 90% of outpatients (Kim et al., 2020). HIRA covers approximately 98% of the overall Korean population and around 80,000 healthcare service providers based on 2011 data (Kim et al., 2020). For this study, the HIRA Paediatric Patient Sample (PPS) dataset for 2018 was used where the estimated extraction proportion is 10% representing 1 million people under the age of 20 (Kim et al., 2020). Both PBS and HIRA databases were provided for research purposes. Table 1 lists each dataset’s qualities (Table 1).

**TABLE 1:** Characteristics of HIRA-PPS and PBS 10% sample databases.

	HIRA-PPS dataset	PBS 10% sample database
Country	South Korea	Australia
Size	∼1 million	∼2.5 million
Sampling	10% random sample	10% random sample
Access	By request	By request
Data availability	2009 till present	2005 till present
Type of administrative database	Reimbursement	Prescription data of PBS-listed medications
Medicine classification	Korean national drug code	PBS item code
Patient classification	Health insurance, medical aid beneficiaries	General, concession, entitlement, doctor’s bag
Information availability		
Deidentified PIN	O	O
Demographic information	O	O (age and gender)
Diagnosis data	O	X
Prescription data	O	O
Hospitalisation data	O	X
Cost data	O	O

Abbreviations: HIRA-PPS, health insurance review and assessment service—paediatric patient sample; PBS, pharmaceutical benefits scheme.

### Study Population

Subjects in this study were defined as paediatric patients aged between 3 and 19 years with a history of more than one asthma preventer dispensing and at least one asthma reliever dispensing in 2018. The Australian PBS 10% population sample dataset does not contain diagnostic codes to identify patients with an asthma diagnosis. Therefore, in order to distinguish patients with asthma, children with dispensing histories of both preventers and relievers were selected. The same method was applied to South Korean subjects. Asthma preventers included ICSs, ICS and LABA combinations, LABAs, LTRAs, cromones, xanthines and biologics. Asthma relievers were defined as salbutamol (SABA) and ipratropium (SAMA).

Additionally, we only included patients aged 3–19 years as access to medicines differed between the countries for children under 3 years of age. In Korea, montelukast and pranlukast (LTRAs), can be used as a preventative medicine in patients aged 2 years or older and zafirlukast, another LTRA can be prescribed in patients aged 5 years or older (Department of Health, 2020). In Australia, montelukast is the only LTRA approved for use in children and is restricted to ages 2–14 years under the PBS (National Health Insurance, 2020). Moreover, in Korea the minimum age for ICS-containing nebules is 1 year and usually 4 years for ICS (Department of Health, 2020). Similarly, in Australia, the minimum age recommended for ICS nebule is 1 year and over and generally 5 years and over for ICS inhalers except for fluticasone propionate which can be used from the age of 1 (National Health Insurance, 2020).

As the study’s primary aim was to examine prescription patterns of asthma preventers, we did not include patients who were only dispensed relievers or oral corticosteroids (OCSs) which are often prescribed for asthma exacerbations, bronchial infections or pneumonia (Masoli et al., 2004; Hon et al., 2014; GINA, 2019; Australian Medicines Handbook, 2020). As mentioned above, our study could not use diagnostic codes, therefore, the inclusion of relievers without the history of preventers could unintentionally encompass patients with conditions other than asthma which could result an overestimation of patients with asthma.

### Statistical Analyses

Descriptive analyses were utilised to characterise demographic factors and preventer medication at baseline for Australian and Korean cohorts. For categorical variables, the number and proportion of variable(s) of interest was calculated using χ^2^ test.

ICS and ICS + LABA groups, were categorised into three groups by strength (low, medium and high dose) based on the Global Initiative for Asthma (GINA) guidelines and the proportion of patients using each preventer group was calculated and compared (GINA, 2015; GINA, 2019).

To compare the ICS and LTRA prescription proportions and prescription proportions by age-groups between Australia and South Korea, the prevalence per 1,00,000 persons was calculated as the number of patients using a specific class of preventer and by age divided by the estimated population of people aged between 3 and 19 in 2018 for each country (Statistics Korea, 2018; Australian Bureau of Statistics, 2019). The standardised prevalence was also estimated.

All analyses were performed using SAS Version 9.4 (SAS Institute) and a *p*-value of <0.05 was considered statistically significant in this study.

## Results

A total of 3,58,470 patients were included in the study. 1,54,200 patients were extracted from the Australian dataset and 2,04,270 patients from the HIRA-PPS dataset of South Korea in the year of 2018.

Table 2 represents the baseline characteristics of the study cohorts. The South Korea cohort was significantly younger than the Australian cohort. In Australia, children aged between 6 and 9 years were most prevalent (31.0%) whereas South Korea had most patients aged between 3 and 5 years (40.9%). South Korea had 70% of their cohort aged nine years or less compared to 56% of the Australian cohort. In both countries, proportions of males were similar and higher than females.

**TABLE 2:** Baseline characteristics of paediatric asthma preventer users in Australia and South Korea, 2018.

Variables	Australia	South Korea	*p* value
	n (%)	n (%)	
Total	1,54,200	2,04,270	
Age group (years)			<0.00001
3–5	38,320 (24.8)	83,570 (40.9)	
6–9	47,760 (31.0)	60,310 (29.5)	
10–12	29,930 (19.4)	22,620 (11.1)	
13–19	38,190 (24.8)	37,770 (18.5)	
Gender			0.10
Male	90,930 (59.0)	1,18,720 (58.1)	
Female	63,270 (41.0)	85,550 (41.9)	
Asthma preventers			<0.00001
ICSs	76,430 (49.6)	10,460 (5.1)	
ICS and LABA combinations	47,080 (30.5)	17,200 (8.4)	
LABAs	130 (0.1)	N/A*	
LTRAs	29,080 (18.9)	1,17,660 (57.6)	
Biologics	80 (0.1)	N/A*	
Xanthines	150 (0.1)	58,950 (28.9)	
Cromones	1,250 (0.8)	N/A*	

Abbreviations: ICS, inhaled corticosteroid; LABA, long acting beta_2_ agonist; LTRA, leukotriene receptor antagonist.

*Data were not available as drugs were not covered by the HIRA.

There were several significant differences in the use of preventer medicines between the two countries (Table 2). Over 80% of Australian children and adolescents were dispensed an ICS preventer (alone or in combination) and 18.8% dispensed a LTRA (100% montelukast). Whereas 13.5% of Korean paediatric patients were dispensed an ICS preventer, 28.9% dispensed controlled release xanthines and 57.6% dispensed LTRAs (44.8% montelukast). Overall, the Australian cohort were prescribed predominantly inhaled preventative therapies (80.1%) and the Korean cohort were dispensed predominantly oral preventative therapies (86.5%) which were a combination of LTRAs and controlled release xanthines (Table 2).

The distribution of strengths dispensed also differed between the countries with a greater proportion of patients dispensed ICS-inhalers or ICS-inhalers in combination with LABA being dispensed higher strengths in Australia than South Korea (10.0% compared to 3.0%). In both countries, among ICS-only inhalers, low dose ICSs were most widely prescribed (69.5% in Australia and 6% in South Korea). Among ICS-inhalers in combination LABA, a medium dose was most frequently used in Australia (53.7%) whereas a low dose was more frequently used in South Korea (91.2%) (Table 3).

**TABLE 3:** Proportion of children dispensed an ICS arranged by strength.

Preventer strength	Australia	South Korea	*p* value
	n (%)	n (%)	
ICS	76,430	10,460	<0.00001
Low dose	53,120 (69.5)	10,010 (95.8)	
Medium dose	20,500 (26.8)	440 (4.2)	
High dose	2,810 (3.7)	<5*	
ICS + LABA	47,080	17,200	<0.00001
Low dose	12,780 (27.1)	15,680 (91.2)	
Medium dose	25,260 (53.7)	680 (4.0)	
High dose	9,040 (19.2)	840 (4.9)	

Abbreviation: ICS, inhaled corticosteroid; LABA, long acting beta_2_ agonist.

*Numbers less than 5 are not displayed, as per the confidentiality policies of the Clinical Practice Research Datalink.

Prescription patterns were analysed by aged groups for the two most prevalent classes of preventers in both countries. ICSs were most widely prescribed in 3–5 years age group in both countries (2,631 per 1,00,000 persons for Australia and 248 per 1,00,000 persons for South Korea). Among ICS and LABA combination inhaler users, prevalence was the highest in patients aged between 13 and 19 years in both Australia and South Korea (1,225 per 1,00,000 persons and 271 per 1,00,000 persons). LTRAs were prescribed the most among 3–5 years in both Australia and South Korea (997 per 1,00,000 persons and 4,012 per 1,00,000 persons) (Table 4).

**TABLE 4:** Prevalence of ICS-based inhalers and LTRAs among different age groups in Australia and South Korea, 2018.

Age group (years)	No. of patients	Population size*	Prevalence per 1,00,000 persons	Standardised prevalence (%)
Australia				
Total	1,52,590	52,47,665	2,905	2.9
ICS				
3–5	25,280	9,60,550	2,631	2.6
6–9	27,750	12,80,613	2,167	2.2
10–12	13,730	9,35,629	1,467	1.5
13–19	9,670	20,70,873	466	0.5
ICS + LABA				
3–5	3,220	9,60,550	335	0.3
6–9	8,400	12,80,613	656	0.7
10–12	10,050	9,35,629	1,074	1.1
13–19	25,410	20,70,873	1,225	1.2
LTRAs				
3–5	9,590	9,60,550	997	1.0
6–9	11,140	12,80,613	868	0.9
10–12	5,830	9,35,629	623	0.6
13–19	2,520	20,70,873	122	0.1
South Korea				
Total	1,45,320	81,91,356	1,774	1.8
ICS				
3–5	3,260	13,16,670	248	0.2
6–9	4,100	18,38,953	223	0.2
10–12	1,690	13,68,538	123	0.1
13–19	1,410	36,67,195	38	0.04
ICS + LABA				
3–5	730	13,16,670	55	0.06
6–9	3,500	18,38,953	190	0.2
10–12	3,030	13,68,538	221	0.2
13–19	9,940	36,67,195	271	0.3
LTRAs				
3–5 years	52,80	13,16,670	4,012	4.0
6–9 years	35,400	18,38,953	1,925	1.9
10–12 years	11,120	13,68,538	813	0.8
13–19 years	18,310	36,67,195	499	0.5

Abbreviations: ICS, inhaled corticosteroid; LABA, long acting beta_2_ agonist.

*The standard age-specific population in 2018 for each age group was used for each country from the Korean Statistical Information Service in the Korea National Statistical Office and the Australian Demographic Statistics in the Australian Bureau of Statistics.

## Discussion

Our cross-sectional study analysing prescribing patterns of asthma preventers in children and adolescents identified Australia’s preference towards ICS (80.1%) as opposed to South Korea which showed a preference towards oral LTRAs (57.6%). Additionally, we found that of the children dispensed ICS the dose used was higher in Australia than in South Korea. Despite the different prevalence in use and dose between the countries ICS users and LTRA users tended to be younger in both countries while ICS plus LABA users tended to be older.

Our Australian results are similar to a previous Australian study by the Australian Institute of Health and Welfare (AIHW) (Australian Institute of Health and Welfare, 2013) which found that among paediatiric patients ICS- preventers were most prevalent followed by LTRAs and cromones (Australian Institute of Health and Welfare, 2013). Also, in line with our results, that study found that the proportion of patients dispensed ICS- in combination with LABA was higher in older children group (12–13 years) whereas as the proportion of ICS only inhalers was higher in younger children group (8–9 years) (Australian Institute of Health and Welfare, 2013). Our results are in line with recommendations for use of LABAs that suggest that the minimum age for use of LABAs in asthma patients is 4 years of age as the safety of LABA use in younger children has not been established (GINA, 2015; Choi et al., 2017; GINA, 2019; Australian Medicines Handbook, 2020). In comparison to previous western studies, Australia displayed similar trends to those of the United Kingdom and Netherlands where ICS-based inhalers were the most frequently prescribed preventer class (Cohen et al., 2007; Uijen et al., 2011; Elkout et al., 2012; Engelkes et al., 2016). This could be due to the similar prevalence of severe asthma in Australia and the United Kingdom where the prevalence is approximately equal to or more than 7.5% requiring the use of inhalers rather than oral formulations (Masoli et al., 2004; Cohen et al., 2007; Elkout et al., 2012; Global Asthma Network, 2018).

Our Korean results were comparable to trends seen in (Sol et al., 2019). A study which utilised Korean National Health Insurance claims from 2010 to 2014 and revealed the most frequent use of LTRAs in paediatric patients with asthma followed by xanthines, ICSs, and ICS and LABA inhalers (Sol et al., 2019). Our study builds on previous Korean study which used the similar dataset and patterns of dispensing by preventer type appear to have not changed over time. Our Korean asthma preventer prescribing patterns are similar to Japanese prescribing trends. In a study conducted by Hamada et al. (2015) LTRAs were most frequently prescribed in school-age children followed by LABAs, ICSs, cromoglycate and sustained release theophylline(Hamada et al., 2015). The preference for LTRAs is likely due to Japanese Paediatric GuideLines (JPGL) which recommend their own stepwise approach for the asthma treatment that is different to GINA or National Asthma Education and Prevention Program (NAEPP) (Lai et al., 2009; Hon et al., 2014; GINA, 2015; Hamada et al., 2015).

Reasons for asthma preventer prescribing differences between Australia and South Korea are not clear from this study, however it is possible that differing cultures, medical systems, asthma prevalence, asthma guidelines and perceptions of safety play a role. Firstly, Korea’s preference towards LTRAs and xanthines is possibly due to Korean culture’s preference for oral medications and customary use of traditional herbal and complementary medicines (Sol et al., 2019; Suh et al., 2017; Chiu et al., 2014). Chiu et al. (2014) conducted questionnaires to assess parents’ perceptions towards asthma treatment in Asia, which found that parents and patients favoured oral tablets because they had perceptions that herbal medicines which are in tablet form, are safer than inhalers leading to lower adherence to inhalers (Chiu et al., 2014; Abu-Shaheen et al., 2016; Lycett et al., 2018). Furthermore, Suh et al. (2017) found that the high LTRA prescription rate in Korea is due to the steroid phobia formed from parents of asthmatic children thinking that the use of corticosteroid would lead to serious adverse events, especially growth impairment (Sol et al., 2019; Choi et al., 2018; Suh et al., 2017). A similar finding is explained in a study which looked into montelukast use over the past 20 years in South Korea (Lee and Kim, 2020). Additionally, another study found that although the efficacy of montelukast in paediatric patients is inferior to that of ICSs, montelukast has no effect on growth unlike ICSs and that montelukast had a similar incidence of ADRs compared to that of the control group (Lee and Kim, 2020). Secondly, the current Korean healthcare system supports medical practitioners to prescribe montelukast. As the number of doctors is comparatively limited in proportion to the number of patients in Korea this indicates the amount of time Korean doctors have to see their patients is restricted (Sol et al., 2019; Suh et al., 2017). In 2017, approximately 2.34 doctors per 1,000 patients was shown in Korea whereas in Australia, 3.5 practicing doctors were available for every 1,000 patients in 2014 (Suh et al., 2017; Choi et al., 2018; Sol et al., 2019). Educating patients and parents of children regarding asthma inhaler techniques can be time-consuming and given the insufficient time provided to medical practitioners, they may favour oral forms of asthma preventers such as LTRAs over ICSs due to the simplicity of administration. Choi et al. (2018) identified that in South Korea, ICS inhalers were more likely to be prescribed in tertiary hospitals than primary health care clinics (84% compared to 21% respectively). In addition, physicians specialising in specialties other than internal medicine prescribed ICS very rarely (Choi et al., 2018). This suggests that asthmatic children with more severe asthma symptoms tend to visit higher grade medical institutions where patients are under the care by specialists in asthma who are more likely to prescribe and educate patients regarding the use of ICS.

Differences in asthma severity could also account for some of the differences seen between countries. According to a study by Hamada et al. (2015), a high prescription rate of LTRAs was noted in Japan indicating that most paediatric patients had relatively mild symptoms which would have required LTRAs or cromones in addition to SABAs as per step 1 therapy of the JPGL (Hamada et al., 2015; Arakawa et al., 2017). This corresponds to the step 2 of GINA (Masoli et al., 2004; GINA, 2015; Global Asthma Network, 2018; GINA, 2019). Differences in asthma severity among countries were shown by Lai et al. (2009) where in the 13–14 years age group, the lower prevalence of 3.8% of severe asthma was seen in Asia-Pacific including South Korea while in English-speaking countries such as the United Kingdom, Ireland and Australia, the prevalence of severe asthma was higher, 7.5% (Lai et al., 2009; Bianchi et al., 2010; Kalayci et al., 2019). A comparable pattern was observed in our results. Among ICS-only users, 13.4% of Australian patients were on a medium dose and 1.8% on a high dose whereas only 0.3% of Korean children were using a medium dose and very few children were on a high dose. In addition, (Suh et al., 2017) suggested that the lower prevalence of severe asthma in Korea is due to a unique Korean national insurance system which covers all residents at low medical costs (Choi et al., 2017; Suh et al., 2017; Choi et al., 2018; Sol et al., 2019). Since montelukast is indicated for the treatment of mild to moderate asthma, South Korea, which has a lower prevalence of severe asthma showed strong inclination of prescribing montelukast and other LTRAs (Lai et al., 2009; Choi et al., 2018; Lee and Kim, 2020). On the other hand, in Australia, asthmatic children may require ICS-based inhalers due to higher prevalence of severe asthma. However, a study by AIHW (Australian Institute of Health and Welfare, 2013), in Australia, found that ICS treatment in children one-third were dispensed high dose ICS which is not in proportion to the prevalence of severe asthma among paediatric patients (Australian Institute of Health and Welfare, 2013) suggesting a possible overuse of high dose ICS among children in Australia (Reddel et al., 2017). Lastly, the difference in asthma treatment guidelines between Australia and South Korea would have influenced prescribing patterns. For instance, for patients aged over 5 years, Korean asthma guidelines recommend a low dose ICS from the step 2 of the preventer therapy where LTRAs or a low dose sustained release theophylline can be used as an alternative (Korean Academy of Asthma, Allergy, and Clinical Immunology, Korean Academy of Allergy and Respiratory Disease, 2016). Australian guidelines suggest to use a low dose ICS from step 2 of the preventer stepped approach where montelukast is recommended as an alternative (National Asthma Council Australia, 2020). Based on the difference in asthma guidelines between two countries, it is plausible to understand Korea’s tendency of prescribing orally available preventers such as LTRAs and theophylline and Australia’s higher rates of prescribing of ICS.

There are some limitations in our study. First, our study concentrated on the prescribing patterns of preventers in paediatric patients with asthma, in those who were on relievers or taking OCSs, therefore we did not include all paediatric patients with asthma. We were unable to use diagnostic codes to select asthmatic children as the Australian PBS data does not contain diagnostic codes. Therefore, we anticipated that inclusion of patients only on relievers or OCSs without preventers would introduce misclassification bias as those patients may be on treatment for respiratory infections and not asthma. This would mean that we would potentially overestimate asthma prevalence in our cohorts (Australian Institute of Health and Welfare, 2012; Australian Medicines Handbook, 2020). Second, our study did not explore all age groups in paediatric patients. Patients aged between 0 and 2 years were excluded from our study as an accurate diagnosis of asthma is often difficult to make in children under the age of 3 and symptoms can be misdiagnosed as pulmonary infections (GINA, 2015; Korean Academy of Asthma, Allergy, and Clinical Immunology, Korean Academy of Allergy and Respiratory Disease, 2016; Global Asthma Network, 2018; GINA, 2019; Australian Medicines Handbook, 2020). Lastly, the number of patients taking montelukast in Australia was likely to have been underestimated for 13–19 years age group since montelukast 10 mg is a private medication, not covered by the PBS in this age group. However, given that the number of patients on montelukast decreased in those aged 10–12 years and the proportion of ICS and LABA combination inhalers gradually increased with age in Australia, it is anticipated that this prescription trend would be similar even if montelukast 10 mg was included in the study.

Despite the limitations described above, this study has important implications for clinical practice and future research regarding ICS and LTRAs, in particular whether the differences in use of LTRAs translates into differences in clinical and safety outcomes between the two countries. A study by Lee and Kim (2020) mentions current guidelines and a Cochrane review that analysed 19 paediatric randomised controlled trials which compared the efficacy of montelukast to that of ICS showed the superior efficacy of ICS (Lee and Kim, 2020). However, both physicians and patients preferred montelukast over ICSs and some patients responded better to montelukast in Korea (Lee and Kim, 2020). Therefore, although many guidelines recommend the use of ICSs over montelukast in paediatric patients with asthma as the first line preventer treatment, future studies are in need that explore prescribing differences noted in asthma preventer medication use between the countries are related to different health and clinical outcomes.

## Data Availability

The data analyzed in this study was obtained from the Australian Government Department of Health (Services Australia) and the Health Insurance Review and Assessment, South Korea. The following licenses/restrictions apply: local legislation. Requests to access these datasets should be directed to nicole.Pratt@unisa.edu.au and/or minsookseo@gmail.com.
