# Supplementary material for: Prescription Patterns of Asthma Preventers Among Children and Adolescents Between Australia and South Korea
Source: Front Pharmacol. 2022 May 20;13:834116. doi: 10.3389/fphar.2022.834116 (PMC9163376; doi:10.3389/fphar.2022.834116)
Supplement: Supplementary file 1 [file DataSheet1.PDF]

Supplementary 1. List of asthma preventers included in the study

Drugs	ATC code	HIRA-PPS (Korea) reimbursement codes[a]	PBS (Australia) reimbursement codes[b]
**ICSs**			
Beclometasone	R03BA01	N/A	8406K, 8407L, 8408M, 8409N,
Budesonide	R03BA02	119530CSI, 119531CSI	2070Y, 2071B, 2072C
Fluticasone propionate	R03BA05	162230CSS, 162231CSS, 162232CSI, 162236CSI, 162233CSI, 162235CSI	8516F, 8345F, 8046G, 8147T, 8148W, 8149X
Fluticasone furoate	R03BA09	500431CSI, 500432CSI	11719T, 11729H
Ciclesonide	R03BA08	497130CSI, 497131CSI	8853Y, 8854B
**LABAs**			
Salmeterol	R03AC12	N/A	8141L
Eformoterol	R03AC13	N/A	8136F, 8239P, 8240Q
**ICS/LABAs**			
Fluticasone propionate/salmeterol	R03AK06	543100CSI, 543300CSI, 544400CSI, 543500CSI, 543200CSI, 543400CSI, 543600CSI	8430Q, 8431R, 8432T, 8517G, 8518H, 8519J

Fluticasone propionate/eformoterol	R03AK11	542900CSI, 542800CSI, 543000CSI	2827T, 10007Q, 10008R
Budesonide/eformoterol	R03AK07	543900CSI, 544000CSI, 543800CSI, 543900CSI, 544100CSI	8796Y, 8625Y, 11273H, 8750M, 11301T, 10024N, 10015D, 10018G
Budesonide/salmeterol	R03AK12	681100CSI, 681000CSI	N/A
Fluticasone furoate/vilanterol	R03AK10	636700CSI, 636800CSI	11124L, 11129R
Beclometasone/ eformoterol	R03AK08	544200CSI	N/A
**LTRAs**			
Montelukast	R03DC03	374603AGN, 374603ATB, 374603ATD, 374601ATB, 374601ATD, 374602ATB, 374602ATD	8627C, 8628D
Pranlukast	R03DC02	216430ASY, 216431ASY, 216432ASY, 216433ASY, 216405ASS, 216406ASS, 216402ASS, 216407ASS, 216401ACH,	N/A

		216408ATB, 216404ATB	
Zafirlukast	R03DC01	249701ATB	N/A
**Xanthines**			
Theophylline	R03DA04	237001ACR, 237003ACR, 237003ATR, 237005ATR	2614N, 8230E, 2634P, 8231F
Aminophylline	R03DA05	107301ATR, 107303ATR	N/A
**Cromones**			
Nedocromil	R03BC03	199701CAE	8365G
Cromoglycate	R03BC01	N/A	8767K, 8334P
**Biologics**			
Benralizumab	R03DX10	N/A	11504L, 11523L, 11529T, 11549W, 11830P, 11847M
Mepolizumab	R03DX09	651201BI	10980X, 10996R, 11003D, 11014Q, 11829N, 11839D
Omalizumab	R03DX05	572902BI, 572901BI	10110D, 10118M, 10956P, 10967F, 11826K, 11835X, 11840E, 11846L, 10109C, 10122R,

		10968G, 10973M, 11824H, 11825J, 11828M, 11864K

Abbreviations: ATC, anatomical therapeutic chemical; HIRA-PPS, health insurance review and assessment service paediatric patient sample; PBS, pharmaceutical benefits scheme; ICS, inhaled corticosteroid; LABA, long acting beta$_2$ agonist; LTRA, leukotriene receptor antagonist.

[a] Data available from the HIRA (http://www.hira.or.kr)

[b] Data available from the PBS (https://www.pbs.gov.au/pbs/)

Supplementary 2. Definitions of 'low', 'medium' and high ICS doses for 6-11 years (GINA)

ICS	ATC (single)	ATC (fixed combination)	Low (μg)	Medium (μg)	High (μg)
Beclometasone dipropionate (CFC)	R03BA01	R03AK08	100-200	>200-400	>400
Beclometasone dipropionate (HFA)	R03BA01	R03AK08	50-100	>100-200	>200
Budesonide (DPI)	R03BA02	R03AK07	100-200	>200-400	>400
Budesonide (nebules)	R03BA02	Not available	250-500	>500-1000	>1000
Ciclesonide (HFA)	R03BA08	Not available	80	>80-160	>160
Fluticasone propionate (DPI)	R03BA05	R03AK11, R03AK06	100-200	>200-400	>400
Fluticasone propionate (HFA)	R03BA05	R03AK11, R03AK06	100-200	>200-500	>500

Abbreviations: ATC, anatomical therapeutic chemical; ICS, inhaled corticosteroid; CFC, chlorofluorocarbon; HFA, hydrofluoroalkane; DPI, dry powder inhaler.

Supplementary 3. Definitions of 'low', 'medium' and high ICS doses for adolescents (GINA)

ICS	ATC (single)	ATC (fixed combination)	Low (μg)	Medium (μg)	High (μg)
Beclometasone dipropionate (CFC)	R03BA01	R03AK08	200-500	>500-1000	>1000
Beclometasone dipropionate (HFA)	R03BA01	R03AK08	100-200	>200-400	>400
Budesonide (DPI)	R03BA02	R03AK07	200-400	>400-800	>800
Ciclesonide (HFA)	R03BA08	Not available	80-160	>160-320	>320
Fluticasone propionate (DPI)	R03BA05	R03AK11, R03AK06	100-250	>250-500	>500
Fluticasone propionate (HFA)	R03BA05	R03AK11, R03AK06	100-250	>250-500	>500
Fluticasone furoate (DPI)	R03BA09	R03AK10	100	Not available	200

Abbreviations: ATC, anatomical therapeutic chemical; ICS, inhaled corticosteroid; CFC, chlorofluorocarbon; HFA, hydrofluoroalkane; DPI, dry powder inhaler.

Supplementary 4. Annual prescription trend of asthma preventers of Australia and South Korea in 2018 arranged by active ingredients

Preventer active ingredient	Australia	South Korea	P Value
	n (%)	n (%)	
	154,200	204,270	
**ICS**	76,430		<.00001
Beclomethasone	950 (0.6)	N/A[†]	
Budesonide	730 (0.5)	360 (0.2)	
Fluticasone propionate	73,110 (47.4)	7,160 (3.5)	
Fluticasone furoate	0	<5*	
Ciclesonide	1,610 (1.0)	2,930 (1.4)	
**ICS+LABA**	47,080		<.00001
Fluticasone propionate and salmeterol	31,560 (20.5)	8,280 (4.1)	
Fluticasone propionate and eformoterol	1,350 (0.9)	700 (0.3)	
Fluticasone furoate and vilanterol	2,920 (1.9)	2,890 (1.4)	
Budesonide and eformoterol	11,200 (7.3)	4,440 (2.2)	
Budesonide and salmeterol	0	0	
Beclometasone and eformoterol	0	890 (0.4)	
**LABA**			N/A[†]
Salmeterol	60 (0.04)	N/A[†]	
Eformoterol	70 (0.05)	N/A[†]	
**LTRA**			<.00001
Montelukast	29,080 (18.8)	91,560 (44.8)	
Pranlukast	N/A[†]	26,100 (12.8)	
Zafirlukast	N/A[†]	N/A[†]	
**Biologics**			N/A[†]
Benralizumab	<5*	N/A[†]	
Mepolizumab	<5*	N/A[†]	
Omalizumab	50 (0.03)	N/A[†]	
**Xanthines**			<.00001
Theophylline	150 (0.1)	7,090 (3.5)	
Aminophylline	N/A[†]	51,860 (25.4)	
**Cromones**			N/A[†]
Nedocromil	500 (0.3)	N/A[†]	

Cromoglycate	750 (0.5)	N/A[†]

Abbreviations: ICS, Inhaled corticosteroid; LABA, Long acting $beta_2$ agonist; LTRA, Leukotriene receptor antagonist.

* Numbers less than 5 are not displayed, as per the confidentiality policies of the Clinical Practice Research Datalink.

[†] Data were not available.
